# Cephalagra and Tic-Douloureux from Accessory Sinus Affection

**Published:** 1900-01

**Authors:** Sargent F. Snow

**Affiliations:** Syracuse, N. Y.


### CEPHALAGRA AND TIC-DOULOUREUX FROM ACCESSORY SINUS AFFECTION.

By SARGENT F. SNOW, M. D., Syracuse, N. Y.

THE sympathy and skill of the medical profession has been well tested in the effort to relieve severe paroxysms of cranial and facial pain. No cases appeal to us more than those afflicted with recurring attacks of cephalagra and tic-douloureux, hence I venture to assume that all medical men have more than a passing interest in those affections.

The many and varied analgesics have been tried and worn out, some leaving their imprints on an already weakened constitution, and some a pernicious habit; some perhaps useful in giving a temporary relief from the distressing pains, but all most unsatisfactory as curative agents, until we have dreaded to see these patients enter our office.

Modern research has brought us to a point where it is now quite generally conceded that there is no pain or neuralgia as a disease *per se*. There must be a primitive cause and it is in the search for these primitive causes that this paper is indited.

Two years ago when the American Rhinological, Laryngological and Otological Society met in Washington,[1] the reader had the honor to present a paper under the title of Headaches from nasal causes,

---

1. Read at the thirty-second annual meeting of the Medical Association of Central New York, at Syracuse, October 17, 1899.

in which thirty cases of the ordinary forms of headaches were reported. Seven claimed to have received relief to the extent of about 40 per cent.; five, 75 per cent.; ten, 90 per cent.; and eight, 100 per cent. from purely nasal work, all pointing most conclusively to the value of this form of treatment.

In this paper I hope to show my reasons for the opinion that we have just as good a right to expect like results in many extraordinary forms where the patients are even thrown into spasms from the severity of the cranial pain, or into the most intense agony by so-called tic-douloureux.

The connection between disordered states of the upper air-passages and cephalalgias is not so debatable now as in 1893, when a paper on Hemicrania and neuralgia,[2] by the author, was brought out. Several essays on headaches by well-known rhinologists have appeared during the past three years,[3] [4] and I expect the future will prove that others can report well authenticated cases of these more intense forms, for which I have chosen the terms cephalagra and tic-douloureux, and, perhaps, have already reported them.

I am not prepared to say that this is the first article calling attention to the relation of pressure within the accessory sinuses to these two troubles; but so far with the limited time and literature at my disposal, I have found no mention of these sources as a probable factor in their production; neither will I presume to say that sinus affections are the only causes of cephalagra and tic-douloureux, but I believe the future will prove that they are determining causes in 75 per cent. of all cases.

It is needless for me to go into detail and show how these reflexes are brought about. You all know the anatomy of the parts I speak of, and the extremely sensitive state a nerve will get into if subjected to severe or constant pressure. This condition of severe and often constant pressure obtains in many of these patients, sometimes from an acute retention of mucus or pus, sometimes from a collection of polyps within the antrum, ethmoid or sphenoidal sinuses.

The reason why collections of mucus, pus or polyps in these cavities cause these most severe paroxysms of headache and tic-douloureux in some, asthma or hay fever in others, and in still others only foul discharges, I will not attempt to explain, but will pass to some of the experiences that led to my present convictions.

About four years ago a relative who had been suffering with neuralgia of the fifth nerve for several months, submitted to an examination of the nose. I expected to find an enlargement sufficient to cause the trouble, but to my disappointment none existed. There was some evidence of sensitive membranes, but no intranasal pressures, and I reluctantly had to refer the case back to the general practitioner. The extreme suffering continued in the manner typical of tic-douloureux until ten months ago when, having had a series of cases exhibiting the same symptoms, in whom I had found ethmoid trouble, I visited the patient with an idea of examining the adjacent sinuses and learned that two weeks previous, following months of acute suffering, there had been a sudden and unexpected relief. An examination at this time showed a nest of small pale tumors that had apparently just broken through the thin wall of bone separating the upper nasal fossa from the ethmoid cavities, and that is exactly what had occurred. The pent up polyps so long hidden had outgrown their habitat, burst its walls, relieving the pressure, and pain had ceased.

In this connection, I will briefly mention two more similar cases that have come under my notice and even now occasionally visit my office. Each of them had had teeth extracted; antimalarial medication, electricity in its various forms, and the best medical care, but with very little good effect, until as a last resort they were referred to a so-called nose specialist. One, a lady who for eight years had suffered in the most severe way, presented interesting features. It was found that the anterior end of the middle turbinate was much enlarged and grown to the septum. The removal of this enlargement was followed by considerable relief, but not the very marked and satisfactory outcome that followed a complete removal of the entire body opening into the ethmoids and clearing out the polyps in the middle and posterior cells.

The other lady, 62 years of age, was referred to me in much the same manner and suffering, if possible, more intensely. In moist weather she would have ten or twelve seizures a day lasting two minutes, and so severe she was compelled to scream and often go into spasms. This condition was continued with slight variation for fourteen years, never free from twitching pains in the face, nutrition had been seriously impaired for a long time, attempts to masticate food would bring on a paroxysm, compelling the frequent use of a feeding tube. Examination showed her face to be pale, moist and shiny, with marked swelling outward of the bridge of nose. The right middle turbinate body was of tremendous size, effectively blocking secretions; this was removed and the ethmoid region opened. The operation was followed by only about 50 per cent. relief, until two months later I entered the posterior cells, curetted the degenerated membranes, and enlarged the sphenoidal opening with most beneficial results.

These abnormal states of the deep sinuses, varying as stated before from simple inflammation of their lining to collection of mucus, pus or polyps, obtain and are the cause of many of our cases of acute hemicrania that persist in some degree after a thorough removal of nasal overgrowths. Endo-ethmoidal or endo-sphenoidal pressures seem also to be a large factor in the cause of the paroxysmal headaches, termed cephalagras, which were mentioned in the early part of this paper.

A detailed recital of cases representing this class might weary you and I will only touch on two of the most extreme that have come to my notice. Both were ladies between thirty-five and forty years of age, giving a history of long and severe suffering from the head pains, so intense at or near the menstrual periods that coma and opisthotonos would supervene. In each there was marked uterine trouble co-existent with an intranasal and sinus affection. In one, the only attempt thus far has been toward an improved pelvic state, which has modified but never prevented the cephalagric seizures.

With the other case the obstructions were removed, good intranasal tone and drainage secured, and while these were maintained, none of the paroxsyms recurred, but with each lighting up of the pelvic inflammation there was marked thickening of the nasal membranes and a strong tendency to headache, which would and did come unless the respiratory tract was kept free and toned up by treatment. Upon the advice of her physician, she submitted to an operation for the repair of a lacerated cervix and the benefit from our combined effort has been marked. He reports that the uterus has become smaller; there is good nasal respiration and drainage at all times with no tendency for headaches.

Such proofs as these, and the marked benefit that follows correction of intranasal states in patients who suffer with those common uterine symptoms, pain or pressure on the vertex, have a strong tendency to make the specialist extravagant in his claims. We don't know but that it will be found that an intermediate, removable nasal factor exists in all headaches (except, perhaps, the occipital), that are associated with pelvic disorders. To be more explicit: It seems that a deranged organic function with congestion, promotes thickening of the nasal membranes, so that surfaces abnormally close press upon each other, causing various reflex disturbances.

Every patient suffering from cephalagra or tic-douloureux does not show an intranasal growth, but, unsuspected, as in the one first mentioned, we may find an ethmoid full of polyps; neither do all cases respond promptly to operative procedures, doubtless because it is almost impossible to reach every region of pressure at one or two sittings. Further than that there may be some areas which we do not wish to meddle with, being of a less chronic order and which can be left for the improvement that follows removal of the worst features. Conservatism is commendable, if not carried too far, but it should be borne in mind, that only slight contact between surfaces may create a profound disturbance where nerves are in such a hypersensitive state.

I would especially emphasise the following observation, as it is one on which may hinge our ultimate success or failure, *i. e.* : Relapses may occur, as it is still possible for neighboring sinuses to become occluded if habits and environment remain unchanged, therefore education in personal hygiene is absolutely imperative, or we may have to be satisfied with modified results.

Digestive disorders, lack of exercise, or a sluggish surface reaction play an important part, and with each of these attacks, symptoms of the old trouble are liable to return; indeed, it is a revelation to see the increased thickness of the membranes, with consequent blocking of secretion, that occurs as the result of these states and no permanent benefit may come from their correction before nasal work, but after nasal work complete success is possible.

BIBLIOGRAPHY.

1. The Medical News, March 10, 1897.
2. The New York Medical Journal, March 31, 1894.
3. Harrison Allen : The Laryngoscope, November, 1896
4. Thompson : Journal American Medical Association, January 14, 1899.

204 EAST JEFFERSON STREET.

